# E2F4 transcription factor is a prognostic biomarker related to immune infiltration of head and neck squamous cell carcinoma

**DOI:** 10.1038/s41598-022-16541-4

**Published:** 2022-07-15

**Authors:** Li Qi, Zihan Ren, Wei Li

**Affiliations:** 1 Department of Otorhinolaryngology, Affiliated Hospital of Inner Mongolia University for the Nationalities, Tongliao, China; 2 Department of Otorhinolaryngology, The First Hospital of China Medical University, 155 Nanjing Street, Heping District, Shenyang, 110001 Liaoning China

**Keywords:** Prognostic markers, Tumour biomarkers

## Abstract

To investigate the relationship between the transcription factor, E2F4, and head and neck squamous cell carcinoma (HNSCC), and to preliminarily explore the signaling pathways and immunological role of E2F4. The mRNA expression of E2F4 in HNSCC was evaluated by searching Gene Expression Omnibus (GEO) and The Cancer Genome Atlas (TCGA) datasets. E2F4 protein expression was analyzed by immunohistochemistry using the CMU1h-ENT database. The association between E2F4 expression and tumor infiltration of immune cells was analyzed. Intracellular signaling by E2F4 was explored using KEGG and GO analysis. The correlation of E2F4 expression with clinical characteristics and its prognostic role were validated and analyzed in TCGA database. From the analysis of GEO and TCGA data, E2F4 expression was found to be up-regulated in HNSCC tumor tissues, and its level was associated with T, Grade, and M staging. Kaplan–Meier curve and Cox analyses indicated that the high expression of E2F4 was related to a poor prognosis. Thus, E2F4 was considered a potential prognostic factor for HNSCC. Immunohistochemical staining showed that E2F4 was mainly localized in the cell nucleus; it was highly expressed in HNSCC tissues, with a significant difference noted from that in pericancerous mucosa tissues. A correlation was observed between the differential expression of E2F4 and the immune infiltration of HNSCC. As revealed by KEGG and GO analysis, differential enrichment was found in the cell cycle, spliceosome, meiosis, microbial polysaccharide synthesis, and WNT signaling pathway, as well as in cyclic adenosine monophosphate, ERBB2, VEGF, GCNP and MYC pathways. E2F4 plays an important role in tumor progression and may be a critical biological prognostic factor for HNSCC. In addition, it functions in the nucleus as a transcription factor, regulates immune cells, and could be a promising molecular target for the diagnosis and treatment of HNSCC.

## Introduction

Head and neck squamous cell carcinoma (HNSCC) is one of the most commonly occurring tumors in the world, with about 550,000 new cases diagnosed every year^1^. Patients with HNSCC usually present with symptoms at an advanced stage, of which approximately 10% develop metastasis^2^. Surgery and adjuvant radiotherapy is the major therapy for local advanced HNSCC but this fails to significantly improve the 5-year survival rate, which seriously affects patients’ quality of life^3^. Therefore, it is necessary to discover highly specific biomarkers of great significance to the development of HNSCC. Such biomarkers may not only provide innovative ideas for early diagnosis and treatment, but may also be used as potential targets in biological therapy for HNSCC.

Many completed studies in recent years focused on the association between the expression of the E2F4 transcription factor in tumor tissues and the clinical characteristics of tumors. E2F4 contains one or more conservative DNA binding domains^4^, binds with target promoters’ transcription factors to regulate target gene expression^5^, and plays a key role in regulating mediated transcription activation^6,7^. *E2F4*, as an oncogene, can promote the occurrence and development of tumors. Most studies have shown that E2F4 expression is higher in gastric and breast cancer tissues than in pericancerous mucosa tissues, and is significantly associated with survival, which indicates a poor prognosis^8^. E2F4 is involved in many cellular and biological processes. For example, it participates in cell proliferation, regulates the cell cycle, and inhibits apoptotic genes^9^. E2F4 expression is abnormally increased in the malignant tumor tissues of gastric, breast, and colorectal cancers^10–12^. However, a study demonstrated that E2F4 expression in breast cancer tissues was lower than that in normal tissues and was unrelated to the tumor staging of patients with breast cancer, which suggests that E2F4 can also act as an anti-oncogene^13^. Thus, E2F4 has a controversial role in tumors; however, in HNSCC, its expression and prognostic role are both unknown.

In the recent years, tumor immunization and treatment have become a research hotspot. CIBERSORT is the latest research tool to evaluate immune infiltration patterns^14^, and is based on high-throughput sequencing data to predict the infiltration patterns of several types of immune cells (e.g., CD8+ T cells, Treg cells, memory T cells, and macrophages) in tumor tissues. At present, CIBERSORT is used to evaluate the immune infiltration of tumor tissues in several malignancies, including liver cancer and HNSCC. Immune infiltration was shown to be related to tumor malignancy and is also a reliable marker for predicting the prognosis of patients with tumors^15–17^. Furthermore, several studies revealed that E2F4 participated in the differentiation and activation of immune cells, and was closely associated with the immune infiltration of malignant tumors^18^; however, its immune effects in HNSCC are yet to be studied.

To uncover any role E2F4 has in HNSCC progression and its relationship with immune infiltration, we conducted the following research: First, we analyzed E2F4 expression in HNSCC and normal mucosal tissues using Gene Expression Omnibus (GEO) datasets in an Oncomine database, and validated this with The Cancer Genome Atlas (TCGA) database; we then analyzed the relationship of the E2F4 level with prognosis. Second, we validated HNSCC (CMU1h-ENT, Department of Otorhinolaryngology, the First Hospital of China Medical University) datasets by immunohistochemistry, and analyzed any correlation with clinicopathological characteristics. Third, we explored the relationship between E2F4 expression and tumor infiltration into immune cells. Finally, by KEGG and GO analysis, we further investigated possible intracellular biological signaling pathways by which E2F4 played a regulatory role.

## Materials and methods

### Data mining and collection

In this study, a GEO (http://www.ncbi.nlm.nih.gov/geo/) dataset (GSE13601) was used to compare the E2F4 expression level between 31 cases of HNSCC tissues and 26 normal tissues. HNSCC data (500 cases of tumor tissues and 44 cases of mucosal tissues) in the TCGA database (http://cancergenome.nih.gov/) were downloaded from the GDC data portal of the National Cancer Institute. All datasets were generated from an RNA-sequencing (Seq) experiment. One hundred and sixty-two cases with a diagnosis of HNSCC were selected from CMU1h-ENT. Paraffin sections of HNSCC and normal mucosal tissues were collected, and the expression characteristics of E2F4 were analyzed by combining the clinicopathological characteristics of patients. All patients gave written informed consent. Experimental data were visualized using R (v.3.4.3) software (http://www.r-project.org/).

### Analysis of E2F4 expression and patient survival

Differences in the expression of E2F4 were analyzed and a box plot for E2F4 expression data was created. The overall survival of HNSCC patients in high- and low-E2F4 expression groups was analyzed, and Kaplan–Meier curves were plotted accordingly. The mRNA expression level of E2F4 in the TCGA–HNSCC database was analyzed with a Limma data package, and its correlation with the clinical characteristics of patients was further analyzed using such clinical parameters as age, sex (male/female), grade (G1–G2/G3–G4), stage (I–II/III–IV), local infiltration (T1–T2/T3–T4), lymph node invasion (N0/N+), and distal metastasis (M0/M1). A “Limma” package in R software was used to identify differentially expressed mRNAs with thresholds of a |log_2_(FC)| > 2.0 and *P* value < 0.05.

### Univariate and multivariate Cox regression analyses

Univariate and multivariate analyses were performed with a Cox proportional hazards regression model. The risk ratio and its 95% confidence interval were calculated. The independent predication values of clinicopathological parameters and E2F4 expression for the survival of patients were quantitatively evaluated, respectively. The prognostic impact of E2F4 on the survival of patients was analyzed by multivariate Cox regression and a forest plot was generated.

### Immunohistochemistry

Paraffin specimens of HNSCC tissues preserved between 2018 and 2020 were selected from the ENT department of CMU1h. Normal pericancerous mucosal tissues were used as a control group. All samples were confirmed by the postoperative diagnosis of two senior pathologists. Pathological diagnostic and TNM staging criteria used were according to the World Health Organization Classification of Head and Neck Tumors (2017). Total patients consisted of 125 males and 37 females, with an age range of 43–72 (58 ± 4) years. All patients received no radiotherapy or chemotherapy before their operation and showed no complications afterwards.

Specimens were fixed with 40 g L^−1^ EDTA solution, embedded in paraffin, and then routinely cut into sections. Immunohistochemical staining was performed with the S-P method. Rat anti-human E2F4 monoclonal antibody was purchased from Abcam plc (Cambridge, UK). All sections were subject to high-pressure antigen retrieval. Immunohistochemical staining was completed according to the kit instructions, followed by development with diaminobenzidine, counterstaining with hematoxylin, and mounting with neutral resin. With confirmed positive HNSCC tissues as positive controls and PBS (instead of primary antibody) as a negative control, the E2F4 expression level was evaluated by immunohistochemical scoring based upon two parameters: (1) Staining intensity: 0 score for negative, 1 score for yellowish, 2 scores for brown, and 3 scores for dark brown. (2) Percentage of positive tumor cells in the total number of cells: 0 score for < 10%, 1 score for 10–25%, 2 scores for 26–70%, 3 scores for > 70%. The multiplication product of the above two scores was the immunohistochemical score, with a range of 0–9 (cases with < 4 or ≥ 4 scores were included in low- or high-E2F4 expression groups, respectively).

### Immune infiltration analysis

The immunological scoring of data was performed with CIBERSORT (https://cibersort.stanford.edu/), and a box plot was then created by combining the differential expression of E2F4. The correlation between the E2F4 expression level and cell subsets (molecule subsets participating in immune infiltration, including B cells, CD4+ T cells, CD8+ T cells, macrophages, neutrophils, and dendritic cells), was analyzed using Tumor Immune Estimation Resource version 2 (TIMER2.0) (http://timer.cistrome.org/) ^19–21^. The log2TPM transformed expression data were used for plotting.

### KEGG and GO enrichment analysis

An ordered list of all specimens related to E2F4 expression was created by Kyoto Encyclopedia of Genes and Genomes (KEGG) and gene ontology (GO), and the expression difference of genes between high- and low-E2F4 expression groups was analyzed. In each round of analysis, 1,000 genome arrangements were completed with the E2F4 expression level used as a phenotype marker. Using a nominal *P* value and normalized enrichment score (NES), we sorted GO and KEGG pathways enriched by phenotype^22^. A false discovery rate (FDR) < 0.05 indicated significant enrichment.

### Ethics approval

All procedures were performed in accordance with the 1964 Helsinki Declaration and its later amendments or comparable ethical standards. This study was approved by the institutional review board (IRB) of the First Hospital of China Medical University (local IRB No. AF-SOP-07-1.0-01). The need for informed consent was waived under the approval of the IRB due to the retrospective design of the study.

## Results

### E2F4 was abnormally expressed in HNSCC and related to clinical characteristics

**Figure 1:**
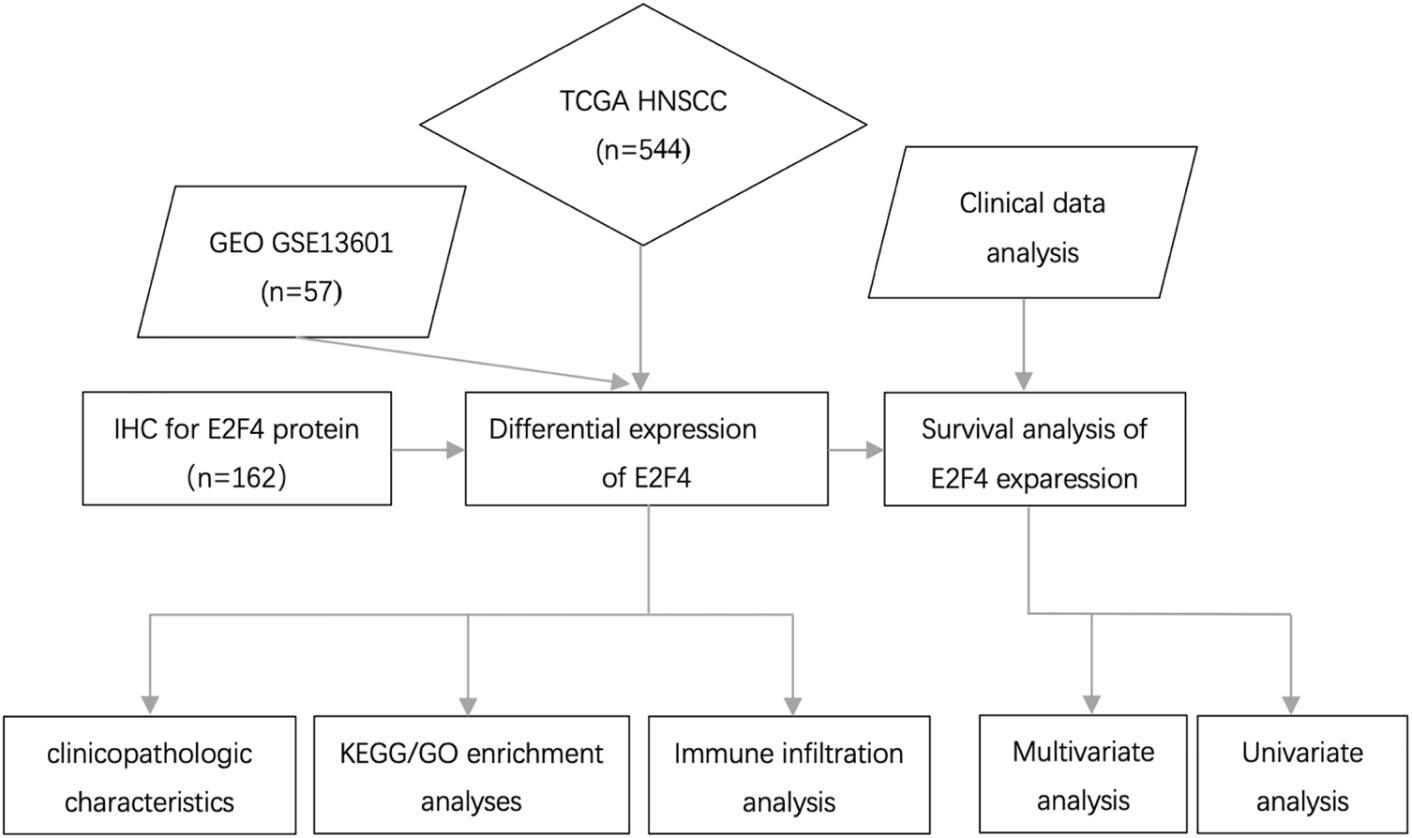
The workflow graph of this study.

**Figure 2:**
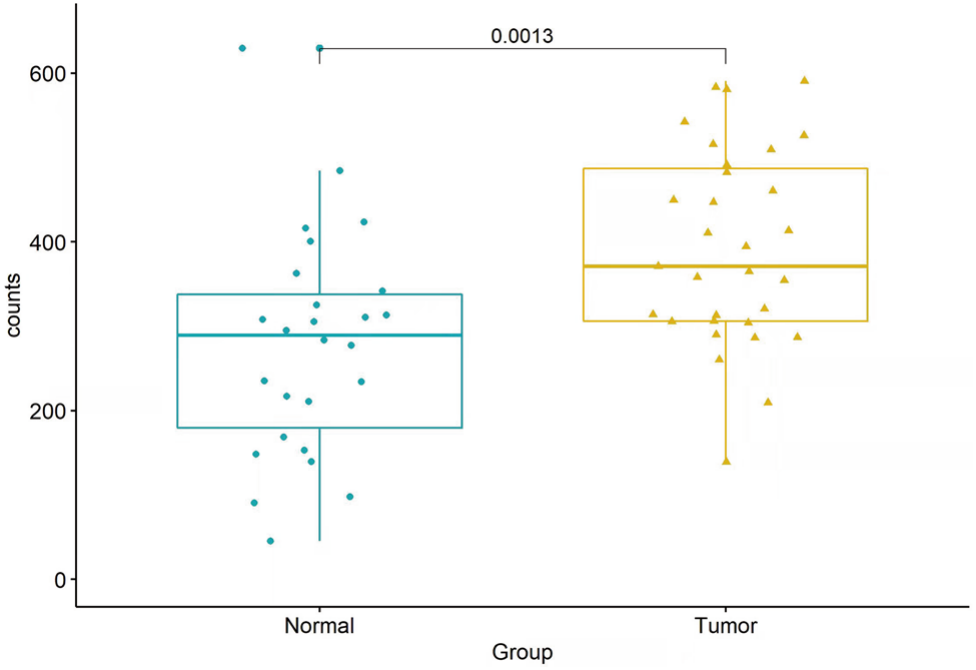
Differential expression of E2F4 in GEO datasets.

**Figure 3:**
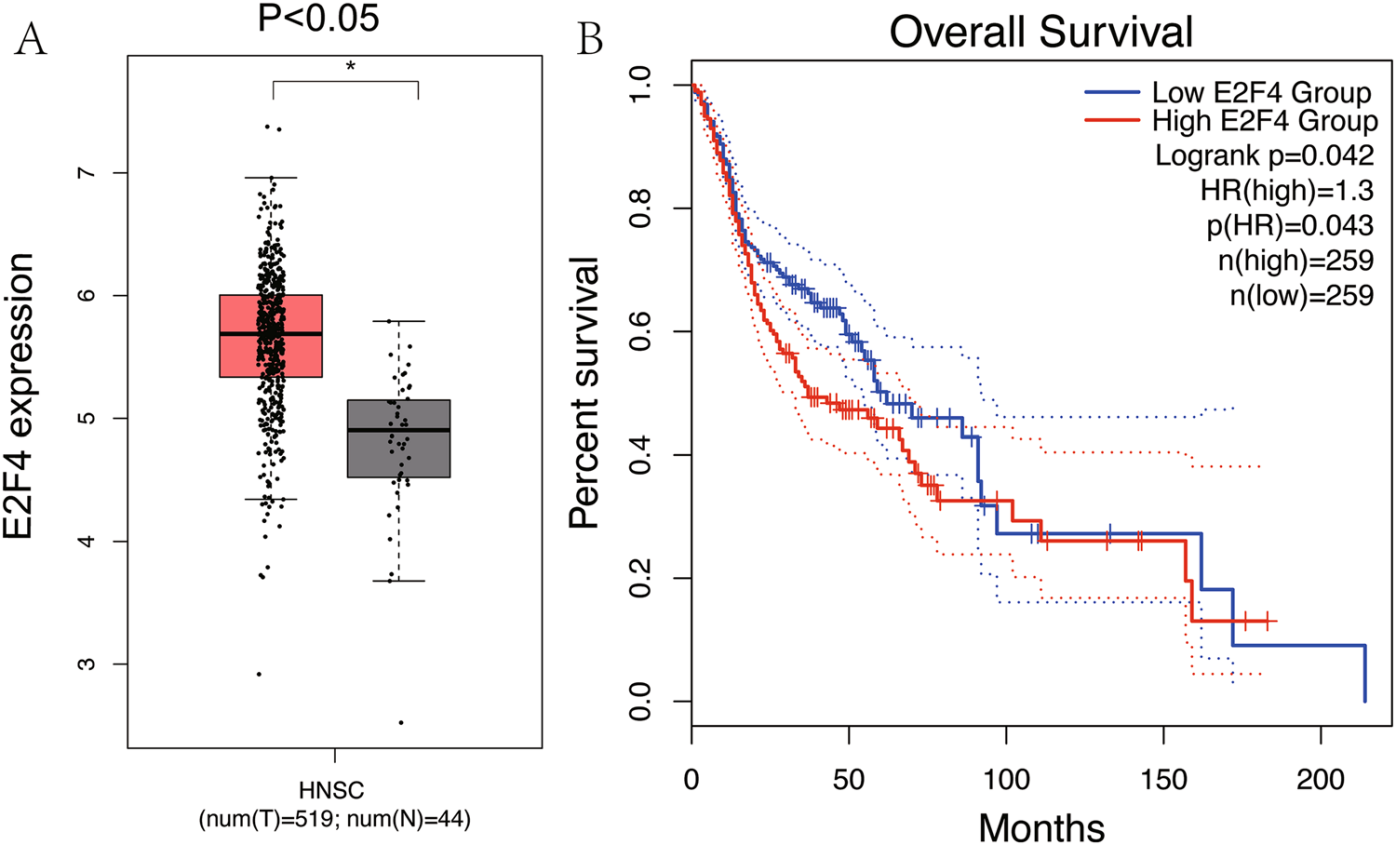
Relationship between E2F4 expression and prognosis of HNSC patients based on the TCGA database. (**A**) Differential expression of E2F4 in HNSC tumor and normal tissues. (**B**) The survival curve for the differential expression of E2F4 was analyzed using GEPIA.

**Figure 4:**
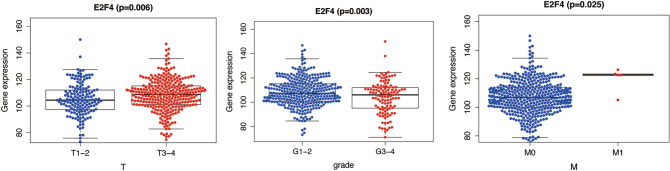
Correlation between E2F4 expression and clinicopathologic characteristics. (**A**) Subgroup analysis of T classification (T1, T2 and T3, T4). (**B**) Subgroup analysis of pathologic tumor grade (grades I, II and grades III, IV). (**C**) Subgroup analysis of M classifications (M0 and M1).

**Figure 5:**
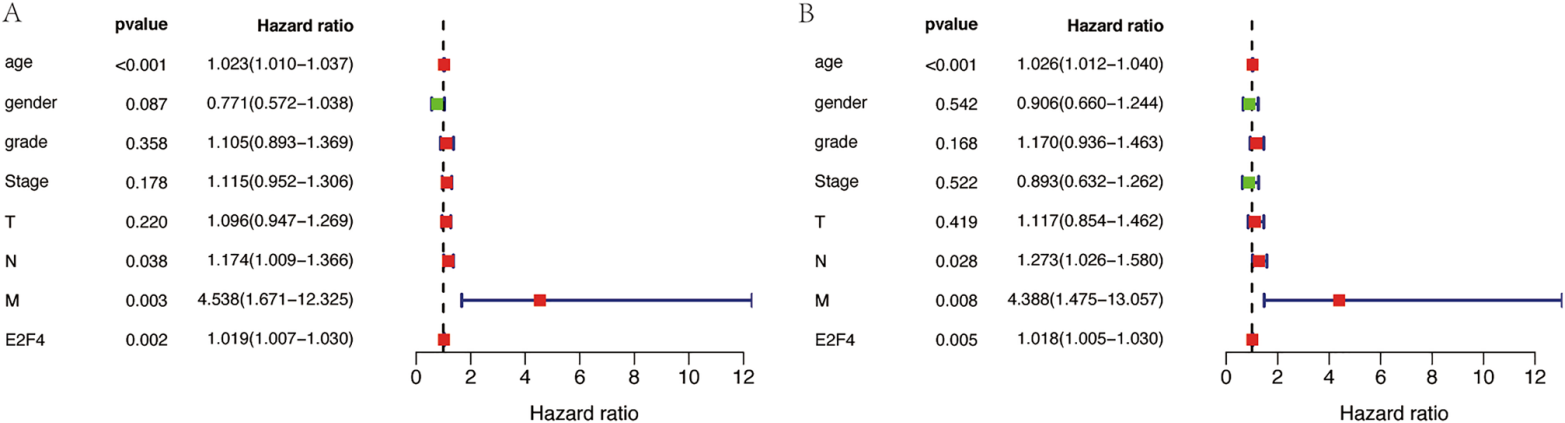
Bioinformatics analysis of E2F4 using the TCGA database (n = 544). (**A**) Univariate analysis of E2F4. (**B**) Multivariate analysis of E2F4. (red indicates positive correlation; green indicates negative correlation. *P* < 0.05).

**Figure 6:**
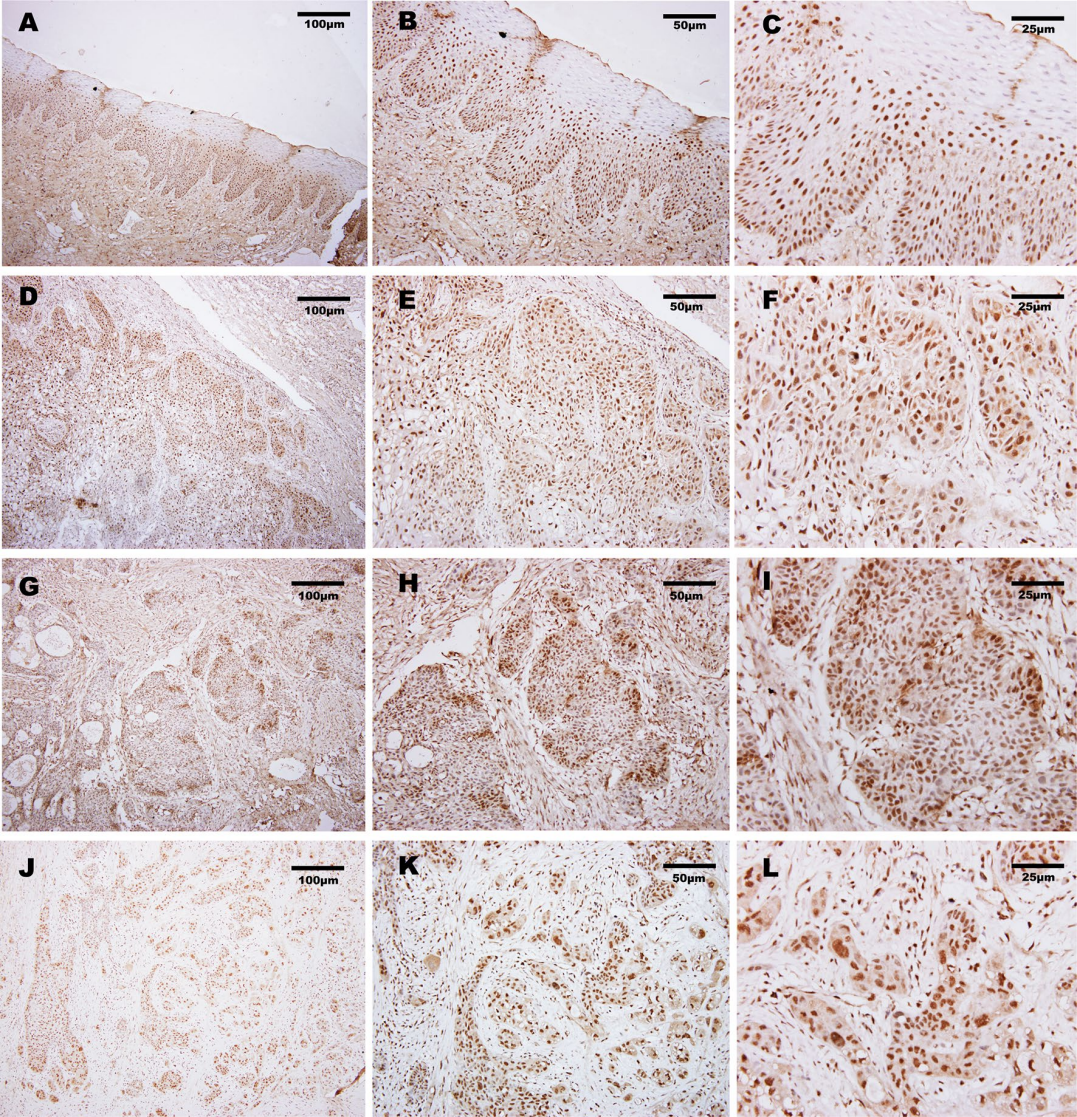
E2F4 is expressed in HNSCC and normal pericancerous mucosal tissues. (**A**–**C**) Low expression of E2F4 in normal pericancerous tissues (×100, ×200, ×400). (**D**–**F**) High expression of E2F4 protein in well-differentiated HNSCC specimens (×100, ×200, ×400). (**G**–**I**) High expression of E2F4 protein in moderately differentiated HNSCC specimens (×100, ×200, ×400). (**J**–**L**) High expression of E2F4 protein in poorly differentiated HNSCC specimens (×100, ×200, ×400).

The workflow graph of this study is summarized in Fig. 1. Based on the GEO dataset “GSE13601”, the E2F4 expression level in tumor tissues was significantly higher than that in normal tissues (*P* < 0.05, Fig. 2). To validate the results, we analyzed E2F4 expression in the TCGA database: the E2F4 expression level was significantly greater in HNSCC tumor tissues than in normal mucosal tissues (*P* < 0.05, Fig. 3A). The survival time of HNSCC patients with high E2F4 expression was significantly shorter than that of HNSCC patients with low E2F4 expression (*P* = 0.042, Fig. 3B). The patients were grouped by age, sex, T stage, grade stage, lymph node metastasis, and distal metastasis. E2F4 expression demonstrated a significant difference among groups of different T stages (T1–2 vs. T3–4, *P* = 0.006), grade stages (grade 1–2 vs. grade 3–4, *P* = 0.003), and distal metastases (M0 vs. M1, *P* = 0.025) (Fig. 4). Univariate and multivariate analyses (HR = 1.013, *P* = 0.028, Fig. 5A; HR = 1.013, *P* = 0.028, Fig. 5B) showed that E2F4 expression was a potential prognostic factor for patients with HNSCC (Table 1). A forest plot was used to visualize results. Furthermore, we validated E2F4 expression with a CMU1h-HNSC dataset, as shown by immunohistochemical staining results. E2F4 mainly localized in the nucleus, and showed markedly higher expression in HNSCC tissues than in normal pericancerous tissues; E2F4 had a high expression rate of 72.04% (67/93) in T3–4 HNSCC, significantly higher than that in of 46.38% in T1–2 HNSCC (32/69; *P* < 0.05, Fig. 6, Table 2). These findings suggest that E2F4 expression was up-regulated in HNSCC tissues, and may be a potential prognostic biomarker for HNSCC.

**Table 1:** Univariate and multivariate correlation analyses of E2F4 in HNSCC patients.

	E2F4 expression		Chi-square value	p-value
	High (n = 93)	Low (n = 69)		
**Age**			0.339	0.561
≥ 65	51	41		
< 65	42	28		
**Gender**			0.443	0.506
Male	70	55		
Female	23	14		
**T**			5.847	0.016
T1 + 2	26	32		
T3 + 4	67	37		
**M**			0.107	0.743
M0	91	68		
M1	2	1		
**N**			3.084	0.079
N0	38	19		
N+	55	50		
**Stage**			2.513	0.113
Stage (I + II)	23	25		
Stage (III + IV)	70	44		
**Grade**			3.481	0.062
Grade (1 + 2)	31	33		
Grade (3 + 4)	62	36

**Table 2:** Correlation between clinicopathologic characteristics and E2F4 expression.

Variables	Univariate		Multivariate	
	HR (95% CI)	P	HR (95% CI)	P
Age	1.023 (1.010–1.037)	< 0.001	1.026 (1.012–1.040)	< 0.001
Gender	0.771 (0.572–1.038)	0.087	0.906 (0.660–1.244)	542
Grade	1.105 (0.893–1.369)	0.358	1.170 (0.936–1.463)	0.168
Stage	1.115 (0.592–1.306)	0.178	0.893 (0.632–1.262)	0.522
T	1.096 (0.947–1.269)	0.22	1.117 (0.854–1.462)	0.419
N	1.174 (1.009–1.366)	0.038	1.273 (1.026–1.580)	0.028
M	4.538 (1.671–12.325)	0.003	4.388 (1.474–13.057)	0.008
E2F4	1.019 (1.007–1.030)	0.002	1.018 (1.005–1.030)	0.005

### Relationship between E2F4 expression and tumor infiltration in immune cells

**Figure 7:**
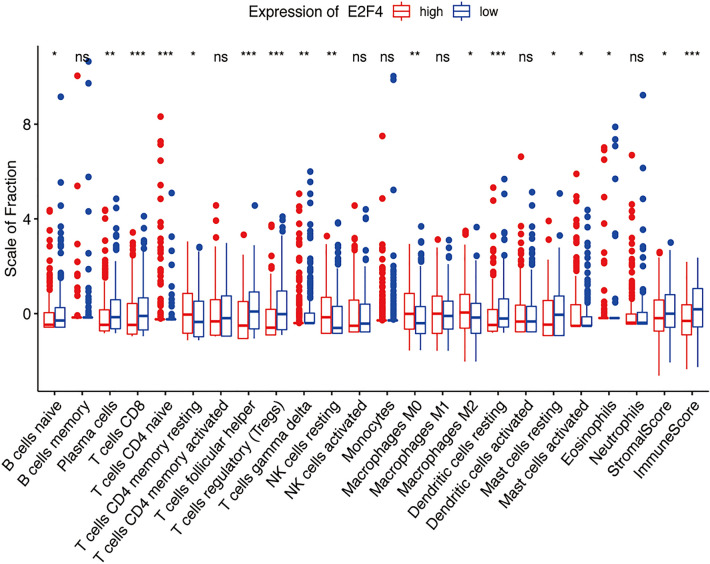
The main immune cells influenced by E2F4 expression included 17 subsets. The number of memory T cells and macrophages (*R* = 0.121, *P* = 0.00741) was increased, while CD8 + T and Treg cells numbers were decreased (*R* = − 0.185, *P* < 0.001; *R* = − 0.232, *P* < 0.001).

**Figure 8:**
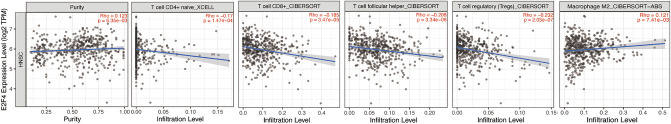
The E2F4 expression level demonstrated a significantly negative correlation with infiltration levels of CD4+ T (*R* = − 0.185, *P* = 1.47e−04), CD8+ T (*R* = − 0.185, *P* = 3.47e−05), Treg (*R* = − 0.232, *P* = 2.03e−07), and T cell follicular helper (*R* = − 0.208, *P* = 3.34e−06) cells, and M2 macrophages (*R* = 0.121, *P* = 7.41e−03) in HNSCC.

Next, we evaluated if E2F4 expression was associated with tumor immune status in HNSCC. E2F4 expression was found to be significantly associated with immune cell infiltration in HNSCC (Fig. 7). The correlation between E2F4 expression and immune infiltration was evaluated using TIMER. In HNSCC, E2F4 was negatively correlated with immune cell infiltration by cells, including CD4+ T (*R* = − 0.185, *P* = 1.47e−04), CD8+ T (*R* = − 0.185, *P* = 3.47e−05), Treg (*R* = − 0.232, *P* = 2.03e−07), and T cell follicular helper (*R* = − 0.208, *P* = 3.34e−06) cells. However, E2F4 expression was positively related to immune purity (*R* = 0.123, *P* = 6.35e−03) and M2 macrophages (*R* = 0.121, *P* = 7.41e−03) (Fig. 8).

### Function and pathways of E2F4 through KEGG and GO analysis

Kyoto Encyclopedia of Genes and Genomes and GO function enrichment analyses were performed, on the basis of the E2F4 expression level, with TCGA data. Significantly enriched signaling pathways were selected according to NES, FDR q values, and nominal *P* values (Table 3). In this study, KEGG enrichment analysis showed differential enrichment in phenotypes with high E2F4 expression such as spliceosome, cell cycle, oocyte meiosis, purine metabolism, and the WNT signaling pathway, as well as enrichment in phenotypes with low E2F4 expression, including linoleic acid and arachidonic acid metabolisms (Fig. S1A). As suggested by GO enrichment analysis, differential enrichment was observed in the following phenotypes with high E2F4 expression: cyclic adenosine monophosphate (CAMP), ERBB2, VEGF, GCNP, and MYC signaling pathways. In addition, enrichment was also found in phenotypes with low E2F4 expression, including kras.lung, kras.prostate, and kras.lung.breast (Fig. S1B).

**Table 3:** KEGG and GO enrichment analysis of E2F4.

Gene set name	NES	NOM p-val	FDR q-val
KEGG_SPLICEOSOME	1.8	0.008	0.381
KEGG_CELL_CYCLE	1.76	0.018	0.302
KEGG_OOCYTE_MEIOSIS	1.81	0.002	0.713
KEGG_PURINE_METABOLISM	1.8	0.004	0.258
KEGG_WNT_SIGNALING_PATHWAY	1.6	0.01	0.326
GO_CAMP	1.7	0.006	0.096
GO_ERBB2	1.59	0.037	0.126
GO_VEGF	1.83	0.004	0.053
GO_GCNP	1.79	0.002	0.053
GO_MYC	1.87	0	0.042
KEGG_LINOLEIC_ACID_METABOLISM	− 1.79	0.016	0.108
KEGG_ARACHIDONIC_ACID_METABOLISM	− 1.95	0	0.042
GO_KRAS.LUNG	− 1.75	0.016	0.202
GO_KRAS.PROSTATE	− 1.65	0.013	0.252
GO_KRAS.LUNG.BREAST	− 1.48	0.041	0.482

## Discussion

In this study, we first explored the relationship between E2F4 expression and clinical phenotype in HNSCC, the role of E2F4 in the progression of HNSCC, especially as a prognostic factor of HNSCC, and its importance as a potential biomarker for the prognosis of HNSCC patients. We also analyzed the correlation of E2F4 expression with the immune-infiltration level of HNSCC in an attempt to find out the interaction between E2F4 and tumor infiltration into immune cells in HNSCC. In addition, we screened E2F4-related HNSCC signaling pathways to understand the potential mechanism of E2F4 in regulating the progression of HNSCC.

We showed that the high expression of E2F4 in HNSCC in the TCGA and GEO database and that this was involved in the occurrence and development of HNSCC. According to several relevant studies, E2F4 showed higher expression in the tumor tissues of breast^23^, colon^24^ and prostate^25^ cancers than in pericancerous tissues. In a study of breast cancer, high expression of E2F4 was observed; such abnormal expression was also associated with TNM staging^11^, which suggests that E2F4 may play a tumor-promoting role in HNSCC, similar to in breast, gastric and prostate cancers.

Kaplan–Meier survival analysis revealed a lower survival rate in HNSCC patients with high E2F4 expression than in those with low E2F4 expression. A worse prognosis in patients with high E2F4 expression was also observed in studies of breast^26^ and bladder^27^ cancers, which indicates that E2F4 is an independent prognostic and predictive factor for the survival of HNSCC patients. Furthermore, a close association of E2F4 with degree of differentiation and T stage of a tumor was found by analyzing the clinical data of patients. Combining the role of E2F4 in colon cancer^28^, it indicates that E2F4 may become a target for the targeted treatment of HNSCC.

The results of immunohistochemistry revealed that the E2F4 protein expression level was high in HNSCC, indicating that E2F4 shows a consistent up-regulation in its mRNA and protein expression levels, and that it participates in the occurrence and development of HNSCC. In in vitro cell experiments, E2F4 inhibited the hypoxia-induced death of isolated ventricular myocardial cells^9^. In in vivo animal experiments, it was found that the high expression of E2F4, detected by in situ hybridization, played an important role in the proliferation and differentiation of mouse epithelial tissues^29^. We believe E2F4 promoted tumor growth by facilitating the proliferation, and inhibiting the apoptosis, of cancer cells and thus enhanced the malignancy of tumor cells. Another study showed that E2F4-specific knockout by lentivirus infection reduced G1/S transition and proliferation rates of normal human intestinal epithelial and colon cancer cells^24^. These findings suggest that E2F4 drives the abnormal cell cycle in tumors and may become a potential target for the molecular treatment of HNSCC.

Immunohistochemical staining showed that E2F4 was mainly located in the cell nuclei of tumor tissues. According to the literature, E2F4 was strongly expressed in the nuclei of cells in several cytological experiments^30–33^. Nuclear expression of E2F4 was similarly observed in the development of breast cancer^34^. We presumed that E2F4 was mainly expressed in the cell nuclei of HNSCC and worked as a transcription factor. Animal experiments have suggested that E2F4 can bind to the p130 promoter region to form a transcription repressor complex and inhibit the transcription of the XPC anti-oncogene via the transforming growth factor-β pathway^35^; this was validated in SCLC^36^. E2F4 is associated with the expression of several downstream oncogenes, including *B-myb*, *rad51,* and *bard1*^37,38^. While *B-myb* is an important regulatory factor for the proliferation, survival, and differentiation of tumor cells^39^, the disturbance of *rad51* and *bard1* is tightly related to DNA repair and cancers^40,41^. We inferred that E2F4 played the role of oncogene as a transcription factor in the cell nuclei of HNSCC.

The tumor microenvironment has a strong influence on the carcinogenesis of HNSCC^42^. Both innate and adaptive (e.g., CD8+ T cells) immune cells play a crucial role in immune surveillance and the control of tumor growth. However, several subsets of immune cells (e.g., macrophages) can also promote tumor growth^43^. Therefore, we aimed to expand our current knowledge regarding the *E2F4* gene in the regulation of the immune response, taking into consideration tumor purity and immunity. We found that immune cells show a high immune cell purity in HNSCC. CD4+ T, CD8+ T, Treg, and T cell follicular helper cells, and M2 macrophages are co-related immune cells, which serve critical roles in HNSCC immune infiltration. Altogether, these data powerfully indicate that E2F4 may be a crucial factor mediating immune-associated pathways. Thus, we suggest that E2F4 may have a potential influence on tumor immunology.

To further investigate the role of E2F4 in HNSCC, we performed KEGG and GO enrichment analyses with the TCGA database. Results showed differential enrichment in the following phenotypes with high E2F4 expression: cell cycle, spliceosome, meiosis, microbial polysaccharide synthesis, and WNT signaling pathway (KEGG enrichment analysis), as well as CAMP, ERBB2, VEGF, GCNP, and MYC pathways (GO enrichment analysis). The association with the cell cycle in KEGG enrichment analysis validated our results, which indicates that E2F4 may be a prognostic indicator and a therapeutic target for HNSCC.

Although this study improved our understanding of E2F4 in HNSCC, there were some limitations. Firstly, this study was designed as a retrospective analysis; therefore, more prospective studies should be performed to verify these results. Secondly, the expression of E2F4 should be verified using cellular experiments. Thirdly, we also cannot clearly estimate the direct mechanisms of E2F4 involved in the development of HNSCC. The specific role of E2F4 in the development of HNSCC should be comprehensively elucidated. Therefore, in the future, a number of experiments will be conducted to demonstrate the mechanistic connections between E2F4 and HNSCC progression.

## Conclusion

In conclusion, these findings indicate that the up-regulation of E2F4 could be a promising molecular target for the diagnosis and treatment of HNSCC. However, the potential role of E2F4 in immune environment regulation and diagnostic function still requires further validation.

## Supplementary Information

Supplementary Figure S1.

## Data Availability

All data generated or analyzed during this study are included in this article.
