# Supplementary material for: E2F4 transcription factor is a prognostic biomarker related to immune infiltration of head and neck squamous cell carcinoma
Source: Sci Rep. 2022 Jul 15;12:12132. doi: 10.1038/s41598-022-16541-4 (PMC9287548; doi:10.1038/s41598-022-16541-4)
Supplement: Supplementary file 1 — Supplementary Figure S1. [file 41598_2022_16541_MOESM1_ESM.pdf]

Supplementary Materials for

E2F4 transcription factor is a prognostic biomarker related to immune infiltration of head and neck squamous cell carcinoma

Li Qi[1,2], Zihan Ren[2], Wei Li[2*]

[1] Department of Otorhinolaryngology, Affiliated Hospital of Inner Mongolia University for the Nationalities

[2] Department of Otorhinolaryngology, the First Hospital of China Medical University

*** Correspondence:**
Dr. Wei Li, Professor
Department of Otorhinolaryngology, the First Hospital of China Medical University
155 Nanjing Street, Heping District, Shenyang
Liaoning Province, 110001
CHINA
Telephone number: 024-83283126
Email address: wli@cmu.edu.cn

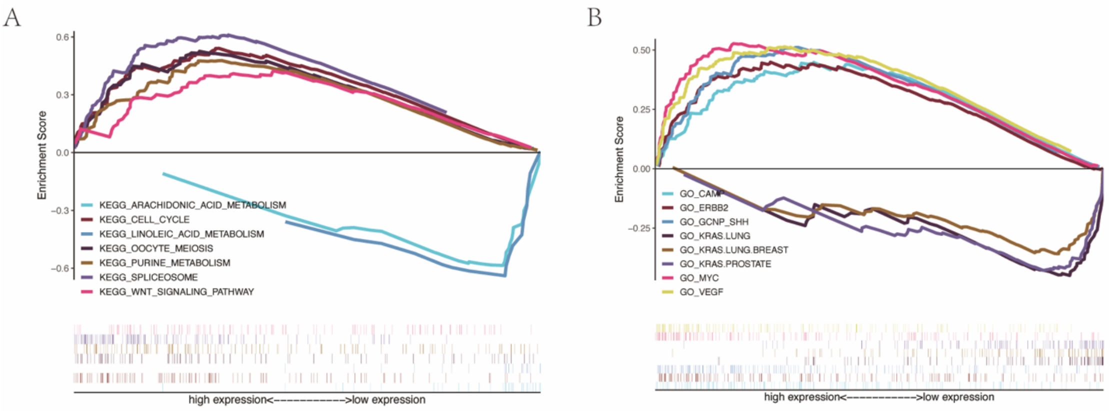

Supplemental Fig. S1. KEGG and GO enrichment analysis. A. KEGG enrichment analysis of E2F4 expression in HNSCC. B. GO enrichment analysis of E2F4 expression in HNSCC.
